# Sepsis and post-sepsis syndrome: a multisystem challenge requiring comprehensive care and management—a review

**DOI:** 10.3389/fmed.2025.1560737

**Published:** 2025-04-08

**Authors:** Jhan Sebastian Saavedra Torres, Francisco Javier Tamayo-Giraldo, Alejandro Bejarano-Zuleta, H. A. Nati-Castillo, Diego A. Quintero, M. J. Ospina-Mejía, Camila Salazar-Santoliva, Isaac Suárez-Sangucho, Esteban Ortiz-Prado, Juan S. Izquierdo-Condoy

**Affiliations:** ^1^Grupo de Investigación en Salud (GIS), Departamento de Medicina Interna, Universidad del Cauca, Popayán, Colombia; ^2^Departamento de Medicina Interna, Universidad Javeriana, Cali, Colombia; ^3^Servicio de Cuidado intensivo Adulto, Clínica Versalles, Cali, Colombia; ^4^Interinstitutional Group on Internal Medicine (GIMI 1), Department of Internal Medicine, Universidad Libre, Cali, Colombia; ^5^Facultad de Ciencias de la Salud, Universidad del Quindío, Armenia, Colombia; ^6^One Health Research Group, Universidad de las Américas, Quito, Ecuador

**Keywords:** sepsis, post-sepsis complications, organ dysfunction, multisystem impact, management and rehabilitation

## Abstract

Sepsis, a medical emergency with high mortality rates, demands comprehensive care spanning from early identification to patient rehabilitation. The sepsis survival chain encompasses early recognition, severity assessment, activation of emergency services, initial antimicrobial therapy, hemodynamic stabilization, and integrated rehabilitation. These interconnected steps are critical to reducing morbidity and mortality. Despite advancements in international guidelines, adherence remains limited, contributing to a significant disease burden. Beyond its acute phase, post-sepsis syndrome (PSS) is characterized by long-term immune dysregulation, chronic inflammation, and metabolic dysfunction, predisposing survivors to recurrent infections, cardiovascular disease, and neurocognitive decline. Mitochondrial dysfunction and epigenetic modifications play a central role in prolonged immunosuppression, impairing adaptive and innate immune responses. Sepsis-induced organ dysfunction impacts multiple systems, including the brain, heart, and kidneys. In the brain, it is associated with neuroinflammation, blood-brain barrier dysfunction, and the accumulation of neurotoxic proteins, leading to acute and chronic cognitive impairment. Myocardial dysfunction involves inflammatory mediators such as TNF-α and IL-6, while sepsis-associated acute kidney injury (SA-AKI) arises from hypoperfusion and inflammation, heightening the risk of progression to chronic kidney disease. Additionally, immune alterations such as neutrophil dysfunction, continuous platelet activation, and suppressed antitumoral responses contribute to increased infection risk and long-term complications. Timely and targeted interventions, including antimicrobial therapy, cytokine modulation, immune restoration, metabolic support, and structured rehabilitation strategies, are pivotal for improving outcomes. However, financial and infrastructural limitations in low-resource settings pose significant barriers to effective sepsis management. Precision medicine, AI-driven early warning systems, and optimized referral networks can enhance early detection and personalized treatments. Promoting public and professional awareness of sepsis, strengthening multidisciplinary post-sepsis care, and integrating long-term follow-up programs are imperative priorities for reducing mortality and improving the quality of life in sepsis survivors.

## 1 Introduction

Sepsis is a multifaceted and potentially life-threatening clinical syndrome arising from a dysregulated host response to infection, characterized by an intricate interplay of inflammation, hypoxia, immune activation, and metabolic reprogramming (1). These systemic disruptions result in profound multisystem dysfunctions, which include significant metabolic derangements affecting the body’s resistance and tolerance strategies (2, 3). Physiological response mechanisms focus on pathogen elimination, often through pathways such as aerobic glycolysis, the production of reactive oxygen species (ROS), the activation of inflammatory cytokines, complement system activation, and the deployment of antimicrobial peptides, which together enhance the immune system’s ability to neutralize and destroy invading pathogens (4). The precise coordination of these adaptive responses is critical for restoring homeostasis, yet this balance is severely compromised during septic scenarios (2, 3).

During sepsis, mitochondrial dysfunction, energy deficits, and reduced ATP/ADP ratios exacerbate clinical prognosis (5). These metabolic disruptions not only amplify organ damage but also undermine immune competence, predispose to immunosuppression and perpetuating a cycle of tissue injury (2, 3). Metabolic and cellular damage induced by sepsis drives the body into a state of massive injury, which proves fatal in a significant percentage of patients, with estimated mortality rates around 38% (6). However, for those who survive the acute phase, the battle is far from over. These patients not only face the challenge of recovering from the initial event but also must contend with long-term complications associated with post-sepsis syndrome (PSS), a condition that remains under-researched but significantly impacts the health and quality of life of survivors (7).

The rising global incidence of sepsis has led to an increasing population burdened by the consequences of PSS, highlighting the urgent need to address both the acute management of sepsis and the chronic repercussions faced by survivors. These include physical, cognitive, psychological, and medical dysfunctions, resulting in diminished quality of life and life expectancy (8). Survivors are at heightened risk of rehospitalization, recurrent infections, and chronic illnesses, with many unable to return to their previous levels of activity or employment (9). Addressing PSS demands a comprehensive, multidisciplinary approach to mitigate its lasting impacts (7, 10).

Early recognition and intervention remain paramount in sepsis management. Strategies such as timely antibiotic administration, appropriate supportive care, and public health measures—including increased vaccination coverage, improved hygiene practices, and judicious antibiotic use—are critical to preventing recurrent infections. For survivors, managing cardiovascular risks through pharmacological treatments, rehabilitation programs, nutritional support, and lifestyle modifications is essential for improving long-term outcomes (10, 11).

This situation underscores the importance of developing strategies not only to reduce mortality rates during the acute phase but also to improve long-term care for sepsis survivors, ultimately minimizing the impact of PSS on their quality of life (12). Addressing the psychological, emotional, and cognitive sequelae of PSS requires well-structured support programs tailored to enhance survivors’ well-being. A personalized, patient-centered approach offers significant potential for delivering comprehensive post-sepsis care. However, further research is urgently needed to refine these strategies and transform the long-term management of sepsis survivors, ensuring a meaningful improvement in their overall quality of life (2, 10, 11).

## 2 Systemic complications of post-sepsis syndrome: cellular and immunological consequences

PSS is a multifaceted condition that affects survivors of sepsis, marked by long-term immune dysfunction, cellular alterations, and systemic complications. A central feature of PSS is persistent immunosuppression, leaving survivors vulnerable to recurrent infections, chronic inflammation, and even malignancies (7, 13). This immune dysfunction is underpinned by alterations in both innate and adaptive immune responses, including impaired leukocyte activation, dysfunctional cytokine production, and reduced antigen presentation (14).

Mitochondrial dysfunction also plays a pivotal role, driving metabolic shifts that exacerbate tissue damage and compromise cellular energy homeostasis (15). These changes, combined with long-term epigenetic modifications in immune cells, contribute to a sustained state of immune dysregulation that impairs inflammation resolution and wound healing (14, 16).

Chronic inflammation, another hallmark of PSS, perpetuates systemic damage and contributes to dysfunction across multiple organ systems, particularly the cardiovascular and musculoskeletal systems (9). Additionally, neurocognitive impairments, such as memory deficits, depression, and anxiety, are common and further compound the long-term burden faced by survivors (17, 18).

Understanding these cellular and immunological disruptions is critical for developing targeted therapeutic strategies to mitigate the chronic impacts of PSS. Interventions focusing on restoring immune function, addressing mitochondrial and metabolic dysfunctions, and controlling persistent inflammation are essential to improve long-term outcomes and the quality of life for sepsis survivors.

## 3 Immune and metabolic dysfunction

Sepsis, a life-threatening systemic condition, induces profound and lasting immune and metabolic dysfunctions that contribute to prolonged immunosuppression in survivors. These alterations include suppression of the antitumoral response, macrophage dysfunction, impaired neutrophil activation, and continuous platelet activation, collectively exacerbating immune dysregulation and increasing the risk of post-sepsis complications.

### 3.1 Suppressed antitumoral response

Prolonged immunosuppression in sepsis survivors is closely associated with an increase in the number and activity of regulatory T cells (Tregs) (19–21). This increase is mediated by chromatin remodeling that promotes the transcription of the regulatory factor Foxp3, enhancing the suppressive capacity of Tregs (21, 22). Additionally, splenic dendritic cells in sepsis survivors facilitate the conversion of Tregs, further suppressing the antitumor activity of CD8^+^ T cells and heightening the risk of tumor growth (23–25).

### 3.2 Reduced IL-10 and CD86 expression and its impact

Sepsis significantly impairs macrophage function, particularly by reducing the expression of costimulatory molecules such as CD86, which are essential for effective antigen presentation and T cell activation (26, 27). This reduction is accompanied by a decline in IL-10 production, a key anti-inflammatory cytokine that plays a crucial role in immune regulation. Together, these deficiencies hinder the immune system’s ability to mount effective responses to subsequent infections, perpetuating post-sepsis immunosuppression and increasing susceptibility to recurrent infections (26–28). These mechanisms present promising targets for therapeutic interventions aimed at restoring immune competency.

### 3.3 Profound alterations in neutrophil function

Neutrophils, vital for microbial control, exhibit significant functional impairments during and after sepsis. Their ability to migrate to infection sites is diminished, compromising effective microbial clearance (29–31). Although sepsis delays neutrophil apoptosis, this prolonged survival does not translate into improved functionality. Neutrophils also exhibit insensitivity to chemotactic stimuli and alterations in IL-10 and CD86 levels. While IL-10 normally helps regulate immune responses to prevent tissue damage, its dysregulation in sepsis exacerbates immunosuppression, further impairing infection control (29, 31). These combined dysfunctions highlight neutrophils as a potential focus for future therapeutic strategies (29, 32, 33).

### 3.4 Continuous platelet activation and its consequences

Persistent platelet activation, triggered by the coagulation cascade, inflammation, and endothelial damage, plays a central role in post-sepsis immune dysregulation. Activated platelets interact with immune cells, pathogens, and endothelial cells, contributing to the formation of microthrombi that compromise microcirculation, exacerbate organ dysfunction, and amplify systemic inflammation (34, 35). Platelets also induce neutrophil extracellular trap (NET) formation, a mechanism designed to trap pathogens but, when dysregulated, can cause excessive tissue damage and inflammation (34, 35).

Furthermore, platelets influence monocyte differentiation into macrophages and modulate macrophage functions, playing a dual role in immune regulation. Persistent platelet activation is closely linked to thrombotic complications and organ failure. Therefore, monitoring and modulating platelet activity represent critical strategies to improve clinical outcomes and reduce mortality in sepsis patients (35, 36).

### 3.5 Natural killer cells in post-sepsis

Natural killer (NK) cells, large granular lymphocytes, play a pivotal role in tumor surveillance and in immune responses to viral and bacterial infections. By producing interferon-γ (IFN-γ), NK cells coordinate early immune responses and enhance the antimicrobial functions of macrophages. However, during sepsis, excessive NK cell activation and overproduction of IFN-γ exacerbate systemic inflammation, contributing to organ dysfunction and physiological damage (37–39).

The biology of NK cells in bacterial infections and sepsis has been extensively studied in murine models, but evidence in humans remains limited. NK cells utilize toll-like receptors (TLR 2/3/4/9) to recognize pathogen-associated molecular patterns (PAMPs), triggering immune activation. IFN-γ secretion by NK cells activates macrophages, promoting phagocytosis and microbial clearance. However, this activation can generate a self-perpetuating feedback loop that amplifies inflammatory responses, exacerbating tissue injury (37, 40).

In the post-sepsis phase, prolonged NK cell activation can shift the immune system toward an immunosuppressive state. Persistent production of IFN-γ and other cytokines disrupts immune homeostasis, diminishing the ability to combat new infections. Furthermore, dysregulated NK cell activity may impair tissue healing and recovery, underscoring their dual role as both mediators of inflammation and immune modulators (37). Targeting NK cell pathways could present new opportunities for mitigating post-sepsis complications and improving patient outcomes.

### 3.6 B-cell subsets in immunosuppression

B lymphocytes are critical for adaptive immunity and play a central role in the immunosuppression observed in sepsis survivors. Sepsis induces significant alterations in the distribution and proportions of specific B cell subsets, which have prognostic implications. Transitional (Tr) B cells and CD5^+^ B cells are markedly reduced in septic patients, correlating with poorer outcomes, while double-negative (DN) B cells are significantly increased and associated with higher mortality risk (41–43).

In non-surviving septic patients, lower proportions and absolute counts of Tr and CD5^+^ B cells are observed during the initial days following hospital admission. Conversely, elevated levels of DN B cells within the first 24 h have been identified as a significant predictor of mortality. These findings highlight the importance of monitoring B cell subsets as prognostic biomarkers for assessing sepsis progression and guiding therapeutic interventions (41, 43).

The functional implications of these alterations are profound. The reduction of Tr and CD5^+^ B cells compromises the generation of effective adaptive immune responses, impairing the ability to combat new infections. Simultaneously, the increase in DN B cells contributes to an immunosuppressive state, elevating the risk of secondary infections and complicating clinical recovery (42, 43). Understanding and addressing these B cell subset alterations could enhance the clinical management of sepsis, offering a pathway toward personalized therapeutic strategies that optimize immune recovery and patient outcomes.

### 3.7 Metabolic shift and immune dysfunction in septic monocytes

Proteomic analyses of blood monocytes in patients with sepsis, septic shock, and post-sepsis have revealed profound metabolic and immunological alterations that impact immune functionality and patient recovery. During the acute phase of septic shock, monocytes undergo a metabolic shift from oxidative phosphorylation to glycolysis, a phenomenon known as the Warburg effect (44, 45). While glycolysis enables a rapid but less efficient form of energy production, this metabolic reprogramming may impair monocyte functionality, limiting their ability to mount effective responses to infections.

In addition to metabolic changes, significant inflammatory dysfunctions have been identified. These include downregulation of major histocompatibility complex class II (MHC-II) proteins and key inflammatory mediators such as mitogen-activated protein kinases (MAPK) and Caspase-1 (44, 46). These alterations reduce monocyte antigen-presenting capabilities and hinder their ability to activate other immune cells, further weakening the host immune response and increasing susceptibility to secondary infections.

During the recovery phase, partial restoration of immune functionality is observed, marked by the upregulation of proteins involved in cytokine signaling pathways, such as interferon-γ (IFN-γ) (44, 46). However, this recovery is often incomplete, leaving patients vulnerable to recurrent infections and other post-sepsis complications. The persistence of these metabolic and immunological disruptions highlights the importance of exploring targeted therapies to fully restore monocyte function and improve clinical outcomes for sepsis survivors (Table 1).

**Table 1:** Overview of cell-specific immune and metabolic dysfunctions in sepsis.

Affected cell	Dysfunction	Key mechanisms	Clinical consequences
Regulatory T cells (Tregs) & CD8^+^ T cells	Suppressed antitumoral response	Increased regulatory T cells (Tregs) suppress CD8^+^ T-cell activity through chromatin remodeling and Foxp3 transcription	Increased risk of tumor growth and reduced immune surveillance in sepsis survivors
Macrophages	Macrophage dysfunction	Reduced IL-10 and CD86 expression impair antigen presentation and immune regulation, increasing infection susceptibility	Weakened immune response to new infections and persistent post-sepsis immunosuppression
Neutrophils	Neutrophil impairment	Defective migration, prolonged survival, insensitivity to chemotactic signals, and IL-10/CD86 alterations weaken immune response	Impaired microbial clearance, increased risk of secondary infections, and prolonged inflammatory dysregulation
Platelets	Continuous platelet activation	Persistent platelet activation contributes to microthrombi formation, endothelial damage, and immune dysregulation	Organ dysfunction, exacerbated systemic inflammation, increased risk of thrombotic complications
Natural killer (NK) cells	NK dysregulation	Excessive IFN-γ production leads to prolonged immune activation, systemic inflammation, and subsequent immunosuppression	Higher susceptibility to new infections, impaired tissue healing, and disruption of immune homeostasis
B lymphocytes	B-cell subset alterations	Reduced transitional (Tr) and CD5^+^ B cells impair adaptive immunity, while increased double-negative (DN) B cells correlate with higher mortality	Poor prognosis in septic patients, increased risk of recurrent infections and immune failure
Monocytes	Metabolic shift in monocytes	Monocytes shift from oxidative phosphorylation to glycolysis (Warburg effect), reducing antigen presentation and inflammatory response	Long-term immune dysfunction, increased infection risk, and incomplete immune recovery

## 4 Multisystemic impact of sepsis

### 4.1 Systemic complications of post-sepsis syndrome: general consequences

PSS is a multifaceted condition that affects sepsis survivors, manifesting as long-term physical, cognitive, and psychological complications. Immune dysfunction is central to PSS, characterized by persistent immunosuppression, heightened infection risk, and an increased likelihood of malignancies (9, 11). Metabolic alterations, driven by mitochondrial dysfunction, exacerbate tissue damage, contributing to chronic fatigue and functional disabilities (9). Chronic inflammation perpetuates systemic damage, affecting multiple organ systems, including cardiovascular and musculoskeletal (8). Survivors frequently report neurocognitive impairments, anxiety, depression, and reduced quality of life, underscoring the profound psychological burden (47). Effective management of PSS requires early recognition, comprehensive rehabilitation, and integrated care approaches to mitigate these long-term impacts, reduce healthcare burdens, and improve survivor outcomes (48).

### 4.2 Senescence and post-sepsis cardiovascular complications

Long-term cardiovascular complications following sepsis, collectively termed post-sepsis syndrome, have emerged as a significant global health concern. These complications include myocardial infarction, acute heart failure, and stroke, with survivors facing an elevated risk of major adverse cardiovascular events (MACE). A central mechanism driving these outcomes is accelerated vascular aging, or premature senescence, which promotes atherothrombosis and exacerbates cardiovascular vulnerability in both sepsis survivors and individuals already at risk for cardiovascular diseases (49, 50).

Premature senescence of endothelial and vascular tissues in sepsis survivors is mediated by several interconnected cellular and molecular pathways, including:

- p53/p21 pathway: Activation of the tumor suppressor p53 induces the expression of p21, an inhibitor of cyclin-dependent kinases (CDKs), halting the cell cycle and triggering cellular senescence.
- p16^INK4a/Rb^ pathway: Inhibition of CDK4/6 activates the retinoblastoma protein (Rb), arresting the cell cycle in the G1 phase and contributing to senescence.
- Oxidative stress: Elevated levels of ROS cause extensive DNA damage, accelerating cellular aging and vascular dysfunction.
- Chronic inflammation: Persistent proinflammatory cytokines, such as interleukin-6 (IL-6) and tumor necrosis factor-alpha (TNF-α), create a senescence-promoting environment that further impairs vascular health (22, 49, 50).

These mechanisms collectively drive vascular dysfunction and contribute to a cycle of chronic inflammation, tissue damage, and cardiovascular decline. Understanding the molecular underpinnings of these processes is crucial for designing effective therapeutic interventions to mitigate the cardiovascular impact of post-sepsis syndrome. Targeting these pathways may provide opportunities to reduce long-term complications and improve the quality of life for sepsis survivors.

### 4.3 Cardiovascular dysfunction in sepsis

Cardiovascular dysfunction is a critical complication of sepsis, extensively studied for over five decades in both clinical and experimental settings. Despite significant research, the underlying mechanisms remain incompletely understood. Patients surviving sepsis often experience persistent cardiovascular, immunological, and cognitive dysfunctions, profoundly impacting their quality of life (38, 51).

While global ischemia is not conclusively established as the primary cause of septic myocardial dysfunction, patients with pre-existing coronary artery disease (CAD) may experience regional myocardial ischemia or infarction during sepsis due to unstable hemodynamics and generalized microvascular dysfunction (51–53). CAD-related ischemia is further exacerbated by the inflammatory and circulatory disturbances characteristic of sepsis (51, 54).

For decades, researchers have postulated the existence of a circulating myocardial depressor factor associated with septic shock. This hypothesis is supported by studies where serum from septic patients reduced the contractility and shortening velocity of rat cardiac myocytes. Notably, these effects were absent in serum from convalescent or nonseptic critically ill patients. Serum ultrafiltrates from patients with severe sepsis have demonstrated cardiotoxicity, linked to elevated levels of interleukins (IL-1, IL-8), complement component C3a, and lysozyme C. Recent findings implicate lysozyme C as a key mediator of cardiodepression in *Escherichia coli* sepsis models, with its inhibition showing potential to prevent myocardial depression (51, 54).

Cytokines play a pivotal role in the pathogenesis of septic myocardial dysfunction. Lipopolysaccharide (LPS) infusion in animal and human models partially reproduces the hemodynamic abnormalities seen in septic shock (38). However, many septic patients exhibit low detectable levels of LPS, suggesting additional mediators contribute to myocardial dysfunction.

Tumor necrosis factor-α (TNF-α), produced by activated macrophages and cardiac myocytes, is an early mediator of septic shock. Although anti-TNF-α therapies have demonstrated transient improvements in left ventricular function, these interventions have not translated into improved survival in clinical trials (51).

Interleukin-1 (IL-1), synthesized by monocytes, macrophages, and neutrophils, also contributes to myocardial dysfunction by impairing cardiac contractility through the stimulation of nitric oxide (NO) production. Additionally, interleukin-6 (IL-6) has emerged as a reliable biomarker of sepsis due to its sustained elevation in circulation, correlating with disease severity and mortality (51, 54).

### 4.4 Impact of sepsis on the brain

Sepsis-induced brain lesions are a significant complication stemming from blood-brain barrier (BBB) dysfunction, neuroinflammation, cerebral hypoperfusion, and the accumulation of amyloid β (Aβ) and tau proteins (55, 56). These pathological changes can be visualized using advanced brain imaging techniques, which reveal white matter alterations and brain atrophy. Addressing these issues requires distinct therapeutic strategies during both the acute and late phases of sepsis. In the acute phase, timely antibiotic use, prevention of ischemic lesions and hypoperfusion, and interventions targeting proinflammatory cytokines are critical. In the late phase, managing neuroinflammation, BBB integrity, Aβ and tau aggregation, and the inhibition of glycogen synthase kinase-3 beta (GSK3β) and receptor for advanced glycation end products (RAGE) pathways are essential (55, 57, 58).

#### 4.4.1 Mechanisms of BBB dysfunction and neuroinflammation

During sepsis, the BBB becomes compromised due to the breakdown of tight junctions between endothelial cells, driven by elevated levels of proinflammatory cytokines such as TNF-α, IL-1β, and IL-6 (55, 57). This disruption allows the entry of inflammatory molecules and toxins into the brain, exacerbating inflammation and neuronal damage (56, 57, 59). Chronic BBB dysfunction interferes with brain homeostasis, fostering sustained neuroinflammation and oxidative stress. These processes contribute to the accumulation of Aβ and tau proteins, hallmark features of neurodegenerative diseases, which further impair neuronal function and promote cognitive decline (55).

#### 4.4.2 Cognitive and neurological sequelae

Post-sepsis, patients frequently experience persistent neurological sequelae, including deficits in memory, attention, and executive functions. These impairments arise from the structural and functional damage caused by neuroinflammation, oxidative stress, and BBB dysfunction during sepsis (56, 58). Additionally, cerebral hypoperfusion during the acute phase contributes to neuronal death and synapse loss, diminishing the brain’s capacity for recovery. This multifactorial damage underscores the long-term burden of sepsis on neurological health.

To mitigate sepsis-induced brain damage and improve long-term outcomes, comprehensive interventions are required. Strategies such as senotherapy, targeting neuroinflammation, and maintaining vascular integrity hold promise. Addressing mechanisms of BBB repair, managing oxidative stress, and developing therapies to prevent Aβ and tau accumulation are critical for improving cognitive outcomes and quality of life for sepsis survivors (55, 57). These approaches highlight the need for continued investigation into the underlying mechanisms of sepsis-induced brain damage and the development of innovative therapeutic strategies.

The complex interplay between inflammatory mediators, microvascular dysfunction, and myocardial depression underscores the need for targeted therapeutic strategies. Further exploration of cytokine modulation and the inhibition of specific mediators, such as lysozyme C, holds promise for reducing cardiovascular complications in septic patients. Understanding these mechanisms can pave the way for improved clinical management and better outcomes for survivors of sepsis.

### 4.5 Recovery and post-sepsis renal challenges

Sepsis-associated acute kidney injury (SA-AKI) is a frequent and serious complication in critically ill patients, significantly contributing to morbidity and mortality. This complex syndrome arises from a combination of macrocirculatory and microcirculatory dysfunctions, mitochondrial impairment, and metabolic reprogramming, which collectively drive its onset and progression (54, 60). Recovery from acute kidney injury (AKI) relies on adaptive mechanisms, such as endothelial repair and tubular cell regeneration. However, maladaptive repair can lead to chronic kidney disease (CKD), increasing the long-term burden on survivors (54).

#### 4.5.1 Pathophysiology and management of SA-AKI

The effective management of SA-AKI hinges on early sepsis recognition and prompt interventions, including appropriate antimicrobials, fluid resuscitation, and vasoactive agents. In severe cases, renal replacement therapy (RRT) is often required to support kidney function. Continuous renal replacement therapy (CRRT) is preferred in hemodynamically unstable patients, although the optimal timing and dosing of RRT remain subjects of ongoing debate (60, 61).

Preventive strategies are critical to reduce the incidence of SA-AKI. Crystalloids are favored over colloids for fluid resuscitation, while norepinephrine is the vasopressor of choice. Central venous oxygen saturation (ScvO2) monitoring has demonstrated superior efficacy compared to lactate clearance or renal Doppler in assessing renal perfusion (60, 62). Long-term complications include the progression of SA-AKI to CKD or, in severe cases, kidney failure requiring permanent renal replacement therapy (KFRT). These outcomes are driven by persistent microvascular damage, chronic inflammation, and metabolic disturbances that impair renal function and exacerbate hypoperfusion and oxidative stress (62, 63).

#### 4.5.2 Role of podocytes in renal recovery

Podocytes, specialized cells in the renal glomeruli, play a crucial role in maintaining intraglomerular pressure and filtration. During sepsis, podocytes are susceptible to damage from systemic inflammation and endothelial dysfunction (64, 65). In response, they activate repair mechanisms, including the regeneration of cellular structures and modulation of basement membrane proteins. Podocytes also release growth factors and cytokines to support the regeneration and repair of glomerular tissue, essential for restoring renal function and minimizing long-term damage (65, 66).

**Figure 1:**
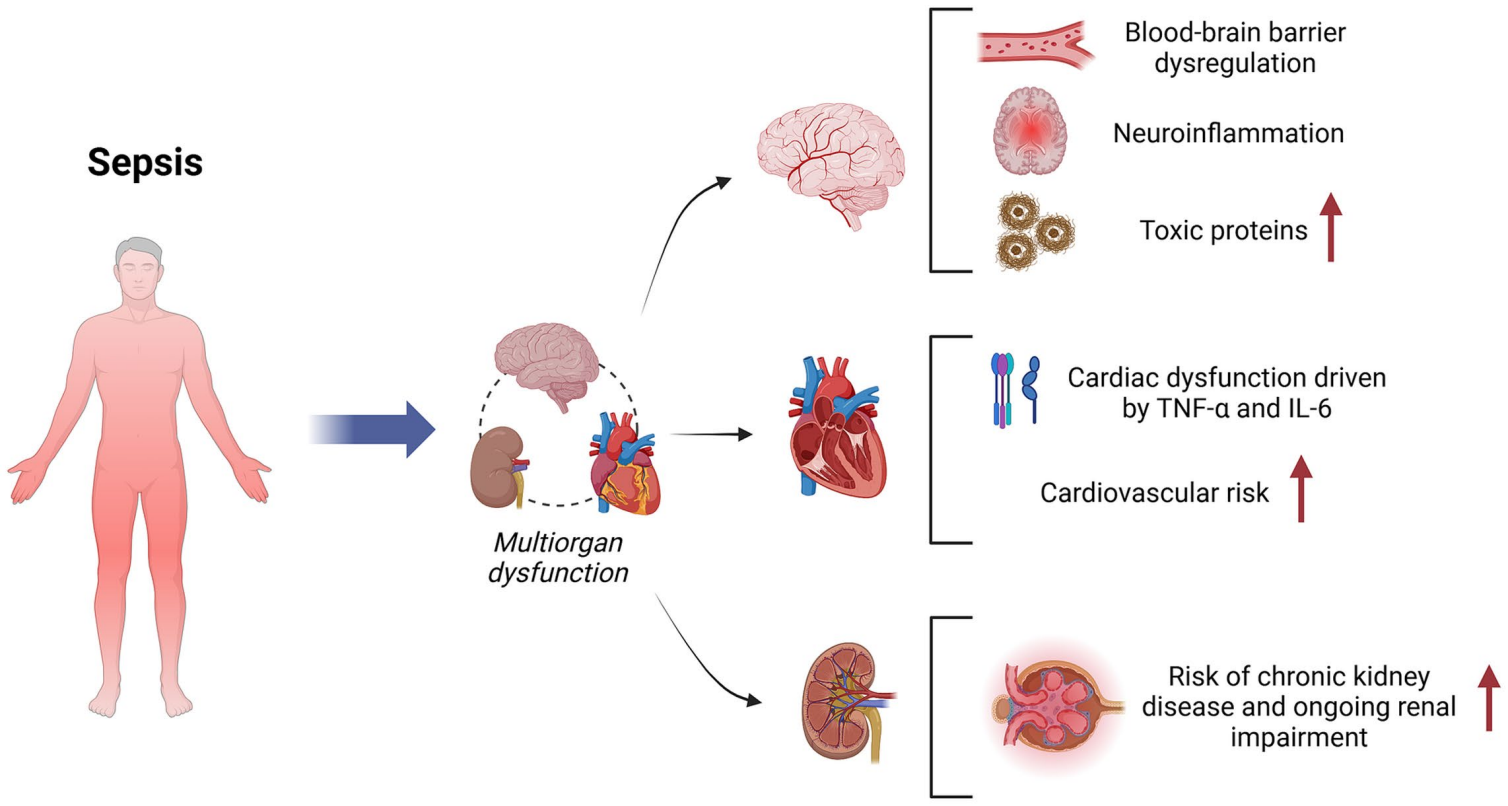
Multisystem impact of sepsis with key pathophysiological insights.

Addressing SA-AKI and its long-term complications requires a comprehensive approach. This includes prevention strategies, timely interventions, and tailored organ-specific support to mitigate renal damage and enhance recovery. Additionally, further research into the molecular mechanisms underlying SA-AKI, including podocyte function and maladaptive repair pathways, is essential. These insights will drive the development of innovative therapeutic strategies to improve outcomes and quality of life for sepsis survivors (54, 61, 64) (Figure 1 and Table 2).

**Table 2:** Sepsis-induced organ impact in cardiovascular, neurological, and renal systems.

System affected	Condition	Mechanisms	Manifestations	Potential interventions
Cardiovascular system	Acute cardiovascular dysfunction	Cytokine storm (TNF-α, IL-1, IL-6), microvascular dysfunction, hemodynamic instability	Hypotension, cardiac arrhythmias, reduced cardiac contractility, septic shock	Early hemodynamic support, vasopressor therapy, cytokine modulation
	Myocardial depressor factors & inflammation	Lysozyme C, IL-1, IL-8, C3a complement activation leading to cardiac depression	Reduced myocardial contractility, persistent heart failure risk	Targeting myocardial depressor factors, cytokine inhibition, myocardial protection
	Post-sepsis cardiovascular complications	Premature vascular aging, endothelial senescence, chronic inflammation, atherothrombosis	Higher risk of MACE (myocardial infarction, heart failure, stroke), increased long-term mortality	Long-term cardiovascular monitoring, anti-inflammatory and vascular repair therapies
Brain (neurological)	Blood-brain barrier (BBB) dysfunction	Breakdown of endothelial tight junctions, allowing inflammatory molecules and toxins into CNS	Cerebral edema, impaired vascular integrity, increased permeability to inflammatory mediators	BBB stabilization, anti-inflammatory therapies, oxidative stress modulation
	Neuroinflammation & cognitive impairment	Sustained IL-6, oxidative stress, hypoperfusion, synapse loss affecting cognitive function	Memory deficits, attention dysfunction, increased risk of dementia	Neuroprotective strategies, synapse preservation, long-term cognitive rehabilitation
	Amyloid β (Aβ) and tau protein accumulation	Neuroinflammation promotes pathological protein aggregation linked to neurodegeneration	Brain atrophy, synaptic dysfunction, worsening cognitive impairment over time	Targeting Aβ and tau pathology, GSK3β and RAGE pathway inhibitors
Renal system	Sepsis-associated acute kidney injury (SA-AKI)	Microcirculatory dysfunction, mitochondrial impairment, metabolic dysregulation	Oliguria, increased serum creatinine, need for renal replacement therapy (RRT)	Early detection, fluid resuscitation, renal protective strategies
	Chronic kidney dysfunction post-sepsis	Persistent inflammation, maladaptive repair, endothelial and podocyte dysfunction	Chronic kidney disease (CKD), renal fibrosis, risk of kidney failure requiring dialysis	Preventive nephrology care, podocyte-targeted interventions, fibrosis control strategies

## 5 Therapeutic perspectives on sepsis and post-sepsis syndrome

Addressing the complexities of sepsis and its aftermath, post-sepsis syndrome (PSS), requires a multifaceted therapeutic approach. Current strategies focus on both immediate intervention and long-term management to mitigate organ dysfunction and improve survivor outcomes (67). Early goal-directed therapy, which includes rapid antimicrobial administration and hemodynamic stabilization, remains a cornerstone of acute care (68). Corticosteroid therapy at moderate doses has shown promise in modulating excessive inflammatory responses, reducing the risk of septic shock progression (7).

Emerging therapies emphasize immunomodulation and metabolic restoration, targeting mitochondrial dysfunction to alleviate immune suppression and chronic inflammation. For example, recombinant human activated protein C has demonstrated potential in reducing coagulation abnormalities and improving endothelial function, though its usage requires further validation (7). Stem cell-based therapies and postbiotics, which aim to restore immune balance and enhance microbiota integrity, are being explored as innovative adjuncts to conventional treatments (69, 70).

The establishment of sepsis follow-up clinics offers a structured approach to identify and address the long-term consequences of PSS, including cognitive, psychological, and cardiovascular complications. These clinics could serve as hubs for personalized rehabilitation plans that integrate physical therapy, nutritional support, and mental health interventions (7).

Continued research into the immunopathology of sepsis is essential for identifying biomarkers and tailoring therapeutic strategies to individual patient needs. Novel approaches, such as targeting immune cell apoptosis and leveraging immunoadjuvant therapies, hold promise for improving survival and quality of life in sepsis survivors. Integrating these advancements into clinical practice could pave the way for a more comprehensive and effective management paradigm for sepsis and PSS.

## 6 Optimizing survival and rehabilitation in sepsis

**Figure 2:**
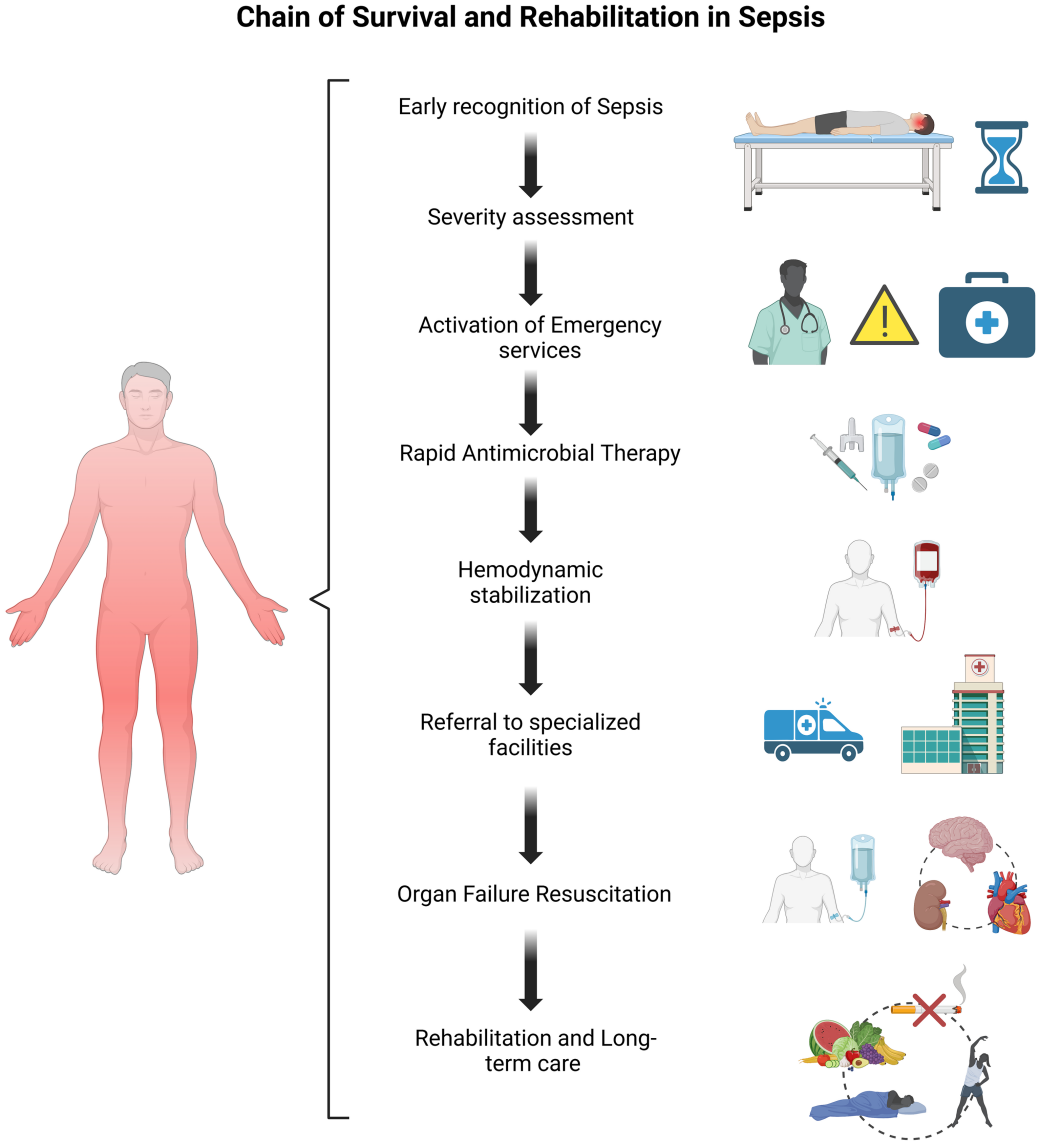
Sepsis survival and rehabilitation chain: key stages from early recognition to long-term recovery.

The chain of survival and rehabilitation in sepsis encompasses every stage of care, from early identification to comprehensive recovery, with the goal of reducing morbidity and mortality. Optimizing each step in this process significantly improves patient outcomes. The key elements of this chain include early recognition of sepsis, accurate severity assessment, timely activation of prehospital emergency systems, rapid initiation of antimicrobial therapy and hemodynamic optimization, appropriate referral to specialized facilities (emergency room, operating room, or intensive care unit), effective organ failure resuscitation, and a robust rehabilitation program. Achieving positive outcomes requires seamless integration and interconnection across these stages (65, 66) (Figure 2).

Despite ongoing updates to international sepsis guidelines, adherence to recommended protocols remains suboptimal, contributing to a significant burden of disease. Variability in healthcare systems, limited resources, and gaps in education and awareness hinder the implementation of standardized sepsis management. Developing and optimizing integrated care networks, spanning prehospital to in-hospital settings, could substantially reduce sepsis-related morbidity and mortality (71, 72).

Timely access to the chain of survival is pivotal in initiating life-saving treatments. Early interventions, such as prompt antibiotic administration and hemodynamic stabilization, are critical to improving survival rates. Equally important is patient referral to appropriate facilities equipped for advanced care. Raising awareness among general practitioners, nurses, paramedics, prehospital caregivers, and the general public is essential to ensuring early detection and rapid response to sepsis. This awareness facilitates timely activation of emergency medical systems and advanced care delivery, irrespective of variations in prehospital service organization (65, 73).

Addressing barriers to guideline adherence and improving the integration of sepsis care pathways are essential for enhancing outcomes. This includes focused education initiatives, enhanced communication among healthcare providers, and the establishment of standardized protocols tailored to specific healthcare settings. Continued efforts to refine and promote the chain of survival and rehabilitation will play a critical role in reducing sepsis-related mortality and improving the quality of life for survivors.

### 6.1 Advances in sepsis diagnosis and treatment

Recent advances in sepsis diagnosis and treatment have emphasized precision medicine, integrating biomarkers, transcriptomic profiling, and novel therapeutic strategies. The identification of immune endotypes, such as SRS1 and IDS, has improved patient stratification, allowing for personalized treatment approaches.

Cutting-edge diagnostic techniques, including next-generation metagenomics (mNGS) and transcriptomic analysis, enable early and accurate differentiation between sepsis and sterile inflammation. Furthermore, targeted therapies, such as immune modulation and metabolic reprogramming, seek to restore immune balance and improve patient outcomes.

The integration of machine learning models into clinical workflows enhances decision-making, enabling more precise antibiotic use while reducing mortality rates. These technological advancements bridge the gap between research and clinical practice, paving the way for more effective, individualized sepsis management (74–76).

### 6.2 Precision biomarkers in sepsis

Despite significant advancements in intensive care management, mortality rates remain high, highlighting the urgent need for more precise diagnostic and therapeutic approaches. The heterogeneity of sepsis has been a major challenge in the development of effective immunotherapies, as patients exhibit divergent immune states ranging from excessive inflammation to profound immunosuppression. To address this variability, recent studies have identified transcriptomic signatures that allow for immune-based patient stratification, enabling a more personalized approach to sepsis management (74–77).

One of the most significant findings in this field is the classification of community-acquired pneumonia into two immune response signatures. The first, known as SRS1, is associated with severe immunosuppression and high mortality, while the second, SRS2, corresponds to a more effective immune response. Furthermore, the identification of an 11-gene panel has demonstrated remarkable accuracy in distinguishing sepsis from sterile inflammation, facilitating a more timely and precise diagnosis (74–77).

At a functional level, sepsis represents an imbalance between antimicrobial response and systemic inflammation. A reduced antimicrobial response coupled with an overactive inflammatory state correlates with higher severity and mortality. This classification surpasses traditional clinical criteria by offering a molecular basis for patient stratification and personalized therapy (74–77).

Beyond these two immune states, recent research has identified a third independent endotype driven by interferon-gamma (IFNγ), termed IDS (IFNγ-driven sepsis). This subset of patients, accounting for approximately 20% of cases, exhibits elevated production of CXCL9, a chemokine with proapoptotic effects that contributes to high mortality. The identification of IDS underscores the potential therapeutic value of targeting IFNγ and CXCL9 to improve patient outcomes (74–77).

Metabolic dysfunction also plays a crucial role in sepsis progression. Macrophage activation-like syndrome (MALS), characterized by extreme inflammation, has been associated with significant metabolic disruption, including alterations in ubiquinone biosynthesis and essential amino acid metabolism. In contrast, immunoparalysis exhibits less metabolic dysregulation, indicating distinct mechanisms of immune dysfunction. The interplay between inflammation and metabolism presents new therapeutic opportunities aimed at modulating the immune response while restoring metabolic homeostasis (74–77).

Recent technological advancements have enabled the development of more precise diagnostic tools, such as next-generation metagenomics (mNGS) and host transcriptomic profiling. By integrating microbial DNA and RNA analysis with immune response assessment, these techniques offer unprecedented capabilities in identifying infections and differentiating sepsis from other systemic inflammatory conditions. The combination of mNGS with machine learning models has demonstrated high diagnostic accuracy, improving sepsis prediction, optimizing clinical decision-making, and reducing unnecessary antibiotic use (74–77).

The integration of transcriptomic signatures, genetic biomarkers, immune stratification, and advanced diagnostic technologies underscores the importance of precision medicine in sepsis management. These advances have the potential to transform clinical practice by improving therapy selection and patient survival. However, the key challenge remains translating these scientific breakthroughs into routine clinical applications to ensure that treatment strategies are tailored to each patient’s unique immunological and metabolic profile (74–76, 78).

### 6.3 Rehabilitation in sepsis: impact and challenges

Rehabilitation in sepsis survivors has emerged as a promising strategy to improve functional recovery and overall quality of life after hospitalization. However, its impact on mortality and other clinical outcomes remains uncertain. Available evidence, including two randomized controlled trials analyzed in a recent systematic review, suggests that rehabilitation interventions in intensive care units (ICUs) may enhance quality of life without significantly reducing ICU mortality, improving muscle strength, or shortening hospital stays. These interventions, which include active or assisted physical therapy and neuromuscular stimulation, are typically administered five times per week in sessions lasting 30 to 60 min, focusing on muscle preservation and mobility enhancement—two key components of post-ICU recovery. Nevertheless, variations in rehabilitation protocols and the limited availability of high-quality data hinder the generalizability of these findings across diverse clinical settings (71, 79–81).

Rehabilitation appears to be most beneficial at two critical stages: during ICU hospitalization, where it may help prevent muscle atrophy and mitigate physical dysfunction, and in the post-hospital phase, where it could support reintegration into daily life and reduce the risk of long-term disability. However, the effectiveness of these strategies is influenced by multiple factors, including the availability of specialized rehabilitation services, healthcare staff workload, and hospital infrastructure. In resource-limited settings, these challenges may further restrict access to early rehabilitation, thereby limiting its potential benefits (71, 79–81).

When compared to rehabilitation approaches in other critically ill populations, existing studies on sepsis survivors underscore the need for more rigorous clinical trials with well-defined methodologies and representative sample sizes. The heterogeneity of rehabilitation protocols, potential selection bias, and the absence of blinding in many studies reduce the strength of the available evidence. To maximize the benefits of rehabilitation, a multimodal approach that integrates physical therapy, nutritional support, and psychological interventions may be more effective than isolated rehabilitation strategies. Despite its potential advantages in functional recovery, the current body of evidence does not yet justify widespread implementation of rehabilitation protocols in sepsis care. Addressing these gaps requires larger, well-controlled studies to establish the efficacy of rehabilitation interventions and to develop evidence-based guidelines for their integration into both hospital and post-hospital care settings (71, 79–81).

### 6.4 Prognostic biomarkers in sepsis

The identification of prognostic biomarkers has transformed the clinical approach to sepsis, allowing for more precise risk assessment and personalized therapeutic strategies. Early detection of organ dysfunction and mortality risk remains a challenge in clinical practice, and the use of complementary biomarkers has the potential to optimize decision-making and improve patient outcomes (82–85).

Among the most promising biomarkers, pentraxin-3 (PTX-3) has emerged as a critical indicator of sepsis severity. As an inflammatory protein involved in the innate immune response, elevated PTX-3 levels correlate with worsening disease progression and higher mortality, particularly when persistently elevated. Its clinical significance lies in its ability to identify patients at a higher risk of septic shock, allowing for more aggressive and timely interventions (82–85).

Another key biomarker is adrenomedullin (ADM) and its stable fragment MR-proADM, both of which serve as sensitive indicators of endothelial damage and microvascular dysfunction in sepsis. Studies suggest that MR-proADM is a more reliable predictor than the SOFA score for assessing disease progression and the need for intensive care, as it correlates with prolonged hospitalization and increased mortality. Beyond its prognostic role, ADM’s association with endothelial dysfunction highlights its potential as a therapeutic target for novel treatment strategies (82–85).

Additionally, endothelial cell-specific molecule-1 (ESM-1), also known as endocan, has been recognized as an emerging biomarker for endothelial dysfunction. By influencing vascular permeability and inflammatory cell activation, it serves as a potential marker for endothelial damage in sepsis. However, its clinical validation remains limited, and further studies are required to determine its reliability in prognostic assessments (82–85).

Dysregulation of the coagulation system is another hallmark of sepsis, and plasminogen activator inhibitor-1 (PAI-1) has been identified as a key mediator in sepsis-associated coagulation disorders. Overexpression of PAI-1 is linked to an increased risk of disseminated intravascular coagulation (DIC) and multiple organ dysfunction, making it a crucial biomarker for assessing severe disease progression. In pediatric patients, specific genetic variants of PAI-1 have been associated with a higher susceptibility to severe sepsis, opening new avenues for research into genetic predisposition and individualized risk assessment (82–85).

Collectively, these biomarkers contribute to a more comprehensive understanding of sepsis pathophysiology, allowing for enhanced risk stratification and tailored therapeutic approaches. Their integration into clinical practice could lead to improved early interventions and mortality reduction. However, their incorporation into standardized protocols requires further validation through multicenter studies to establish their clinical reliability and effectiveness (82–85) (Table 3).

**Table 3:** Overview of the prognostic biomarkers in sepsis.

Biomarker	Physiological role	Clinical utility	Prognostic implications	Future directions
Pentraxin-3 (PTX-3)	Inflammatory protein involved in the innate immune response	Identifies high-risk patients for septic shock and mortality	Persistent elevation correlates with higher mortality and severe inflammation	Standardization needed for clinical integration
Adrenomedullin (ADM)/MR-proADM	Regulator of endothelial function and microvascular homeostasis	More reliable predictor of disease progression than SOFA score	Reflects endothelial dysfunction, prolonged ICU stay, and increased mortality	Requires validation as a therapeutic target
Endothelial cell-specific molecule-1 (ESM-1)/endocan	Marker of endothelial dysfunction and vascular permeability	Potential marker for endothelial damage, though clinical validation is limited	May help assess vascular integrity but requires further study	Limited data on its reliability in clinical settings
Plasminogen activator inhibitor-1 (PAI-1)	Mediator of coagulation and fibrinolysis in sepsis	Associated with increased risk of DIC and multi-organ dysfunction	Genetic variants may predispose pediatric patients to severe sepsis	Potential role in personalized medicine needs further exploration

### 6.5 Challenges in adherence to international guidelines and barriers in sepsis care

Adherence to international sepsis guidelines is hindered by multiple challenges, including variability in healthcare provider training, limited access to advanced diagnostics and therapeutics, and resistance to changes in clinical practice. The heterogeneous clinical presentation of sepsis and the difficulty in early recognition further contribute to inconsistencies in implementing standardized recommendations. Additionally, in many healthcare settings, excessive workload and insufficient infrastructure create significant obstacles to the effective application of evidence-based protocols (86, 87).

Efforts to enhance guideline adherence focus on several key strategies. Continuous healthcare provider training remains essential to improve early recognition and protocol compliance. The implementation of clinical decision-support tools and early warning systems powered by artificial intelligence has demonstrated potential in identifying high-risk patients and optimizing timely interventions. Additionally, the standardization of protocols across healthcare institutions, coupled with periodic audits and feedback mechanisms, has proven effective in reinforcing compliance with established recommendations (86, 87).

Resource-limited settings face additional constraints, including financial barriers and infrastructural deficiencies, which significantly impact sepsis management. Optimizing care in these environments requires targeted interventions, such as training programs focused on early sepsis identification, prioritization of cost-effective treatments—notably timely antibiotic administration and fluid resuscitation—and strengthening referral networks and telemedicine capabilities. Furthermore, integrating public health initiatives that enhance sepsis prevention and expand access to essential treatments can help reduce disease burden and improve patient outcomes in these settings (86–88).

## 7 Conclusion

Sepsis is a life-threatening medical emergency characterized by multisystem dysfunction, impacting on critical organs such as the brain, heart, and kidneys. It is associated with significant morbidity and mortality. Early identification and timely intervention are essential to improving clinical outcomes and minimizing complications, including cognitive, myocardial, and renal dysfunction. Despite advancements in international sepsis guidelines, adherence remains insufficient, underscoring the need for optimizing care networks and enhancing awareness among healthcare professionals and the public.

The cornerstone of sepsis management lies in early interventions, including prompt antimicrobial therapy and hemodynamic support, to stabilize patients and mitigate organ damage. Beyond acute care, the establishment of integrated networks and comprehensive rehabilitation programs is crucial to improving long-term outcomes and quality of life for sepsis survivors. Continued efforts to promote education, enhance adherence to guidelines, and refine therapeutic strategies are necessary to address the challenges posed by this complex condition.
